# The Effect of Spice Powders on Bioactive Compounds, Antioxidant Activity, Phenolic Components, Fatty Acids, Mineral Contents and Sensory Properties of “Keşkek”, Which Is a Traditional Food

**DOI:** 10.3390/foods11213492

**Published:** 2022-11-03

**Authors:** Mehmet Musa Özcan

**Affiliations:** Department of Food Engineering, Faculty of Agriculture, Selcuk University, 42031 Konya, Turkey; mozcan@selcuk.edu.tr; Tel.: +90-332-2232933

**Keywords:** Keşkek, bioactive properties, polyphenols, fatty acids, minerals, sensory properties

## Abstract

“Keşkek”, which is a dish of Western Anatolia, Thrace, the Eastern Anatolia Region, the Black Sea and Central Anatolia, is a traditional dish made mainly of split wheat and meat—although it varies according to the regions in Anatolia—which is usually made at weddings and holidays. In this study, the effects of thyme, coriander and cumin spices on the fat content, bioactive properties, phenolic component, fatty acid composition, mineral contents and sensory properties of “Keşkek” were investigated. The oil yields of “Keşkek” types were determined to be between 14.90 (control) and 21.20% (with cumin). Total phenolic and flavonoid contents of “Keşkek” types’ added spices were established as between 7.02 (control) and 77.10 mg/100 g Gallic Acid Equivalent (GAE) (with thyme) to 20.24 (control) and 132.14 mg quercetin equivalent (QE)/100 g (with thyme), respectively. Moreover, the antioxidant activity values of “Keşkek” samples varied between 0.04 (control) and 2.78 mmol Trolox Equivalent (TE)/kg (with thyme). Among these phenolic constituents, gallic acid was the most abundant, followed by catechin, rutin and 3,4-dihydroxybenzoic acid, in descending order. Oleic and linoleic acid contents of the “Keşkek” oils were detected between 25.51 (with thyme) and 30.58% (with cumin) to 38.28 (with cumin) and 48.49% (control), respectively. P, K, Mg and S were the major minerals of “Keşkek” samples. Considering the sensory characteristics of the “Keşkek” samples, “Keşkek” with thyme was appreciated, followed by “Keşkek” with cumin and “control and Keşkek” with coriander in decreasing order.

## 1. Introduction

“Keşkek”, which is a dish of Western Anatolia, Thrace, the Eastern Anatolia Region, the Black Sea and Central Anatolia, is a traditional dish made mainly of split wheat and meat—although it varies according to the regions in Anatolia—which is usually made at weddings and holidays. One of the traditional dishes that the Yörük culture brought to Turkish cuisine in particular, “Keşkek” is among the indispensable tastes of gastronomy in Turkey in summer and winter. The most important traditional dish of Yörük and Turkmen weddings, “Keşkek” has ceased to be a wedding meal in Turkey and has become one of the foods consumed daily. Since it is a meal made from wheat, making “Keşkek”, which makes you feel full for a long time after you eat it, is just as difficult [1]. The “Keşkek” tradition was included in the United Nations Educational, Scientific and Cultural Organization, Representative List of the Intangible Cultural Heritage of Humanity as of 2011 [2]. In the history of humanity, food can be described as an important cultural factor in the formation of national identity as well as the most basic need of individuals [3,4]. Local food is defined as food or beverage that is produced, grown in that location, and has a local identity and characteristics specific to the region. Local cuisines create a competitive environment for entrepreneurs in many countries’ tourism markets and tourist destinations [5]. The most important factor that determines the food cultures of the countries is geographical conditions. Later, cultural experiences and transfers from generation to generation affected the general culture and culinary culture [6]. It has been reported that spices and herbs are an excellent source of phenolic compounds with good antioxidant activity, as well as being used as flavor additives in foods [7]. Coriander, turmeric and ginger, rich in antioxidant compounds, are the most important herbs and spices used to improve the functional profile of bread [8]. It has been reported that bread from wheat flour with added coriander leaf powder has been appreciated for its high antioxidant value, moisture-holding capacity, efficient baking features and sensory properties such as fragrance aroma and taste [9]. The purpose of the use of spices in food products is to give taste, flavor and color, to protect foods and to provide durability. Spices alone are not staple foods and are rich in flavor, odor or color substances, and the compounds that give this feature are the essential oils in their structure. Spices used to flavor foods have been known for thousands of years [8,10]. So far, no detailed study has been found to determine the bioactive properties, phenolic components, fatty acids, mineral contents and sensory properties of “Keşkek” with spice added. The aim of the current study is to investigate the effects of thyme, coriander and cumin spices on the fat content, bioactive properties, phenolic component, fatty acid composition, mineral contents and sensory properties of “Keşkek”.

## 2. Materials and Methods

### 2.1. Material

After mixing 500 g of wheat with potable water for 10 min, the seed was crushed in a mortar for 15 min and the pericarp was separated from the seed. It was then sieved and separated from the seed pods. Then, seeds without pericarp were soaked in water for 12 h. Then, by soaking in water, the wheat was boiled until it burst and turned into mush. Towards the end of the boiling, 50 mL of sunflower oil, 75 g of butter and 5 g of salt were added and it continued to be cooked for 5 minutes on low heat. A total of 1% g of thyme, coriander and cumin were added separately to each 100 g of hot “Keşkek” and mixed well. Each of the obtained “Keşkek” was air-dried. No spice was added to the control group.

### 2.2. Material and Methods

#### 2.2.1. Moisture Content

After the “Keşkek” samples were ground, the moisture amounts of the “Keşkek” types were determined in an oven (Nüve FN055 -Ankara-TURKEY) at 105 °C until a constant weight [11].

#### 2.2.2. Oil Content

A total of 10 g of air-dried and ground “Keşkek” samples was placed in a Soxhlet cartridge and infused with petroleum ether. Afterwards, extraction was carried out with petroleum ether at 50 °C for 6 h. After the extraction was completed, the solvent in the micella was removed by evaporator at 50 °C. The remaining crude oil was calculated gravimetrically (%) [11].

#### 2.2.3. Extraction Procedure

Extraction process of “Keşkek” samples was conducted according to method stated by Abreu et al. [12]. After 15 mL of methanol: water (80:20 *v*/*v*) was added to 1 g ground “Keşkek” sample; the mixture was held in an ultrasonic water bath for 30 min. After this time, the samples were centrifuged at 443.52 g for 10 min. The supernatant obtained was separated in a membrane filter. After 10 mL n-Hexane was added to this supernatant, it was stirred with vortex. Methanol phases were collected at each step. Then, after the samples were evaporated at 50 °C, the extracts obtained were dissolved in 15 mL methanol.

#### 2.2.4. The Content of Total Phenolic

Total phenolic contents of “Keşkek” extracts were determined by using the Folin–Ciocalteu reagent [13]. A total of 1 mL of Folin–Ciocalteu was added to extract and stirred by vortex for 5 min. At the end of this period, after 10 mL 7.5% Na_2_CO_3_ solution was added to the mixture, it was stirred. The final volume of sample was completed with 25 mL distilled water. After the samples were kept in the dark for 1 h, the total phenol amounts of the samples were recorded at 750 nm with a calibration curve made using gallic acid (0–200 mg/mL) as the standard. Results obtained in triplicate were described as mg gallic acid equivalent/100 g (fw).

#### 2.2.5. The Contents of Total Flavonoid 

Total flavonoid contents of caper extracts were determined using colorimetric method according to Hogan et al. [14]. The extract of “Keşkek” (1 mL) was stirred with 0.3 mL of NaNO_2_, 0.3 mL of AlCl_3_ and 2 mL of NaOH, respectively, and kept in dark for 15 min. The absorbance of mixture was recorded at 510 nm using spectrophotometer (Shimadzu-Japan). The results obtained are described as mg quercetin (QE)/100 g [14].

#### 2.2.6. Radical Scavenging Activity of “Keşkek” Samples

The radical scavenging activity values of “Keşkek” extracts were determined by using 1,1-diphenyl-2-picrylhydrazyl (DPPH) according to method stated by Lee et al. [15]. After 2 mL methanolic DPPH solution was added to extract, the mixture was vigorously stirred and kept at room temperature for 30 min. At the end of this period, the absorbance was recorded at 517 nm. The results were given as mmol Trolox (TE)/kg. Antioxidant activity (%) was calculated using following relation:

Inhibition (%) = Conrol (Absorbance 517) − Extract (Absorbance 517)/Control (Absorbance 517) × 100

#### 2.2.7. Determination of Phenolic Compounds

The phenolic compounds of “Keşkek” extracts were identified via Shimadzu-HPLC equipped with PDA detector and Inertsil ODS-3 (5 µm; 4.6 × 250 mm) column. Acetic acid (0.05%) (A) and acetonitrile (B) mixtures were used as mobile phase. The flow rate of the mobile phase was 1 mL/min at 30 °C. The injection volume was 20 µL. The peaks were obtained at 280 and 330 nm with PDA detector. The total running time was calibrated as 60 min. Gradient elution was performed for separation and a mixture of 0.05% acetic acid in water (A) and acetonitrile (B) as the mobile phase was used. The flow rate of the mobile phase was 1 mL/min at 30 °C, and the injection volume was 20 µL. The peaks were recorded at 280 and 330 nm using a PDA detector. The total running time per sample was 60 min. Phenolic compounds were determined according to the retention time and absorption spectra of peaks of standard compounds (Figure S1). The total area under peak was used to quantify each of the phenolics.

#### 2.2.8. Fatty Acid Composition

The fatty acid methyl esters (FAMEs) of “Keşkek” oils were determined by gas chromatography GC-2010 (Shimadzu-Japan) consisting of flame-ionization detector (FID) and capillary column [16]. For the fatty acid analysis of “Keşkek” oils, the carrier gas and total flow rate were nitrogen with a flow rate of 1.51 mL/min and 80 mL/min, respectively. Furthermore, the split rate was also 1/40. The temperature of the injection block and detector was calibrated to 260 °C. In addition, column temperature was calibrated at 120 °C for 5 min, increased to 240 °C at 4 °C/min and held at 240 °C for 25 min [11]. The identification of peaks was performed by comparing the retention times obtained with methyl ester standards used.

#### 2.2.9. Mineral Contents of “Keşkek”

After “Keşkek” was dried at 70 °C, each “Keşkek” sample was ground in a laboratory-type mill. About 0.5 g ground “Keşkek” was burned by using 5 mL of 65% HNO_3_ and 2 mL of 35% H_2_O_2_ in microwave system. After adding 20 mL of distilled water up to its volume, minerals in “Keşkek” were measured by Inductively Coupled Plasma Atomic Emission Spectroscopy (ICP-OES) (Varian-Vista, Ottoway, Australia) [17]. 

#### 2.2.10. Sensory Evaluation of “Keşkek”

Hedonic test was chosen to analysis of sensory properties (appearance, color, texture, flavor and general appreciation). Sensory analyses were performed under white light in sensory test rooms with special cabins. The control group was taken as the standard and the treated samples were compared with the control. For the sensory analysis of “Keşkek” samples, experienced staff at Selçuk University were chosen. For this purpose, 8 educated panelists were asked to provide numerical values. Evaluation of sensory properties of appearance, color, texture, flavor and general appreciation of the “Keşkek” samples was carried out the next day by a panel of minimum ten semitrained judges on five-point hedonic scale (1—very bad, 2—bad, 3—middle, 4—good, 5—perfect) [18].

### 2.3. Statistical Analyses

Analysis of variance of results was made by JMP version 9.0. The results are mean ± standard deviation of independent “Keşkek” types [19].

## 3. Results and Discussion

### 3.1. The Chemical and Bioactive Compounds of “Keşkek” Samples

The chemical properties, bioactive compounds and antioxidant activity values of “Keşkek” enriched by several spice (thyme, coriander and cumin) powders are presented in Table 1. Chemical and bioactive properties of “Keşkek” samples showed some fluctuations depending on the spice types added. While the moisture contents of “Keşkek” samples vary between 5.70 (with coriander) and 7.15% (control), the oil contents of “Keşkek” samples were determined to be between 14.90 (control) and 21.20% (with cumin). Total phenolic and flavonoid contents of “Keşkek” types’ added spices were established as between 7.02 mg GAE/100 g (control) and 77.10 mg GAE/100 g (with thyme) to 20.24 mg QE/100 g (control) and 132.14 mg QE/100 g (with thyme), respectively. Moreover, the antioxidant activity values of “Keşkek” samples varied between 0.04 mmol TE/kg (control) and 2.78 mmol TE/kg (with thyme). While the moisture contents of “Keşkek” with the spices decreased compared to the control, the oil amounts of the “Keşkek” samples partially increased according to the control. The highest oil content was determined in “Keşkek” with cumin, followed by thyme and coriander spices, in descending order. Spices increased the total phenolic and flavonoid contents and antioxidant activity values of “Keşkek” samples compared to the control (*p* < 0.05). According to this situation, a linear relationship was observed between the total phenolic amounts and antioxidant activity values of “Keşkek” samples (Figure S2). The highest total phenol, total flavonoid and antioxidant activity values were detected in “Keşkek” sample with thyme. It was thought that the high bioactive properties of "Keşkek" with thyme might be due to the phenolic components found in high amounts in thyme. Spices, which are a potential source of natural antioxidants, have many phytochemical compounds such as phenolic diterpenes, flavonoids and phenolic acids [20]. Thyme, coriander and cumin contained 7.78 μg CE/g and 14.25 μg QE/g, 9.22 μg CE/g and 3.38 μg QE/g, and 10.17 μg CE/g and 101.34 μg QE/g total phenolic and total flavonoids, respectively [21]. Kozłowska et al. [22] determined 28.07 g GAE/kg total phenol in herbal extract of coriander plant. Cumin and coriander extract contained 7.0 and 4.2 mg GAE/g total phenol [23]. Antioxidant activity values of cumin and coriander extract were established as 1.48 and 2.2 mg/mL, respectively [23]. The antioxidant properties of phenolic acids and flavonoids are due to their redox properties, their ability to chelate metals and their quenching of singlet oxygen [24]. According to the literature, coriander, cumin and thyme contain high levels of total phenols and total flavonoids and have high antioxidant activity values. It has been observed that the addition of these spices to the “Keşkek” increases the bioactive properties of the “Keşkek”. Differences in the bioactive properties of “Keşkek” samples may have resulted from the ingredients and applied processes, such as boiling conditions and the temperature applied.

### 3.2. The Phenolic Compounds of “Keşkek” Samples

**Figure 1:**
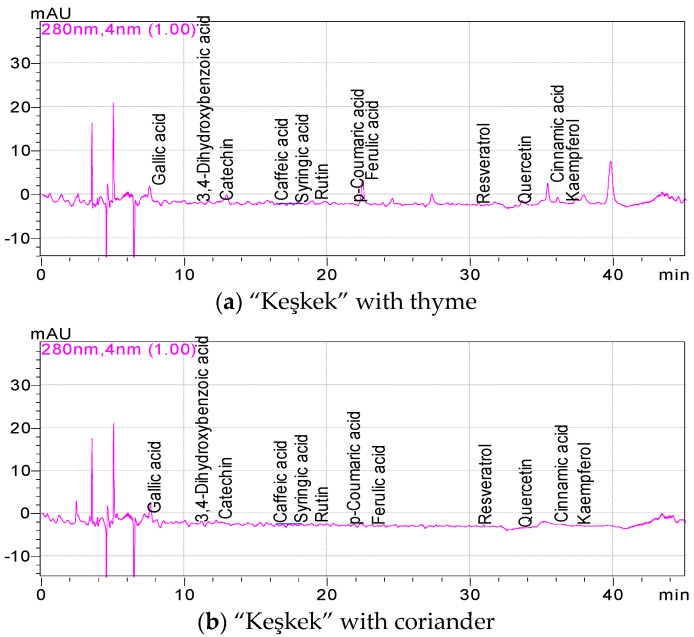

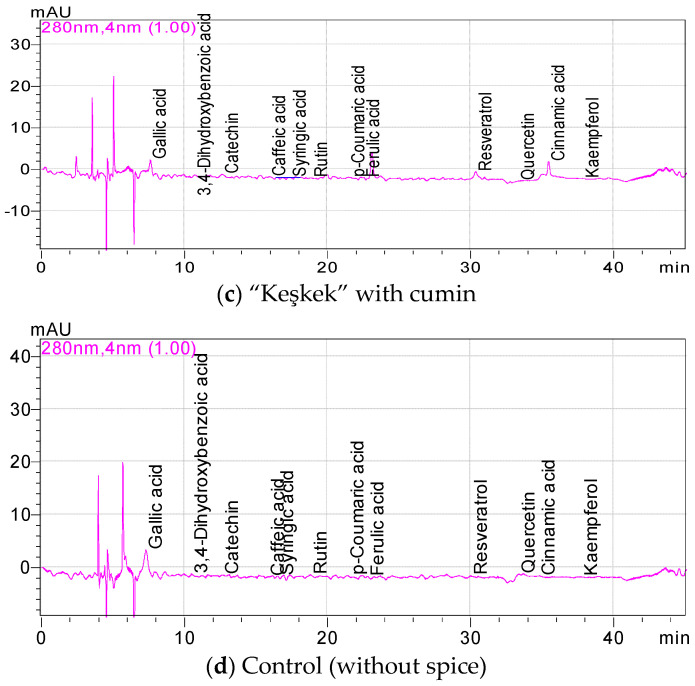
Phenolic chromatograms of control and “Keşkek” samples (**a**–**d**) with spice added.

The phenolic constituents of “Keşkek” samples with thyme, coriander and cumin spices are shown in Table 2. The phenolic components of the “Keşkek” samples were determined between by the HPLC. Gallic acid, 3,4-dihydroxybenzoic acid, catechin and rutin were the dominant phenolic constituents of “Keşkek” samples (Figure 1). Among these phenolic constituents, gallic acid was the most abundant, followed by catechin, rutin and 3,4-dihydroxybenzoic acid, in descending order. While gallic acid contents of untreated (control) and treated “Keşkek” samples with spices change between 25.97 (control) and 50.53 mg/100 g (with cumin), catechin contents of “Keşkek” types were identified between 2.54 mg/100 g (control) and 6.52 mg/100 g (cumin). In addition, rutin and 3,4-dihydroxybenzoic acid contents of “Keşkek” samples made by adding spices were detected between 1.95 mg/100 g (with cumin) and 4.33 mg/100 g (with thyme) to 0.40 (control) and 3.12 mg/100 g (with cumin), respectively. Moreover, quercetin amounts of “Keşkek” samples were recorded between 0.47 (with cumin) and 1.32 mg/100 g (with thyme), while ferulic acid amounts of “Keşkek” samples are determined to be between 0.21 (with coriander) and 1.91 mg/100 g (with thyme). The phenolic components (except *p*-coumaric acid, resveratrol and kaempferol) of the “Keşkek” sample made with the addition of thyme increased significantly when compared to the control. The gallic acid, 3,4-dihydroxybenzoic acid, catechin and caffeic acid contents of the “Keşkek” sample prepared with the addition of coriander increased significantly compared to the control and the amounts of other components decreased. In addition, the phenolic components of “Keşkek” samples prepared with cumin were significantly increased when compared to the control (except for syringic acid, rutin, *p*-coumaric acid, quercetin and kaempferol). Accordingly, the “Keşkek” sample with thyme added was the most phenolic compound, followed by the “Keşkek” sample with cumin and coriander in decreasing order. Kozłowska et al. [22] determined 2.693 g/kg chlorogenic acid, 1.045 g/kg ferulic acid, 0.114 g/kg caffeic acid, 0.562 g/kg rosmarinic acid, 0.552 (+)-g/kg caffeic acid and 18.414 g/kg rutin in herbal extract of coriander plant. In previous study, methanolic extracts of coriander seeds contained high chlorogenic and caffeic acids [25]. Methanol extract of cumin and coriander contained 3.30 and 3.10 μg/g vanillic acid, 2.34 and 2.78 μg/g 4-coumaric acid, 76.51 and 155.96 μg/g chlorogenic acid, 1.86 and 1.15 μg/g ferulic acid, 1.04 and 4.10 μg/g catechin, 7.97 and 5.25 μg/g naringin, 1.14 and 17.89 μg/g quercetin and 17.94 and 6.30 μg/g kaempferol [23]. It is understood that the phenolic components of the spices added to the “Keşkek” are high in the literature data. Therefore, it was observed that the addition of spices in the “Keşkek” increased the phenolic components significantly compared to the control group. In addition, it can be said that “Keşkek”, which has gallic acid, catechin, rutin and quercetin content, can be considered as a functional food in terms of human health. The differences in the phenolic component amounts of the “Keşkek” samples may have been caused by the chemical structure of the spices used and the heat treatment applied.

### 3.3. Fatty Acid Profile of the “Keşkek” Oils

**Figure 2:**
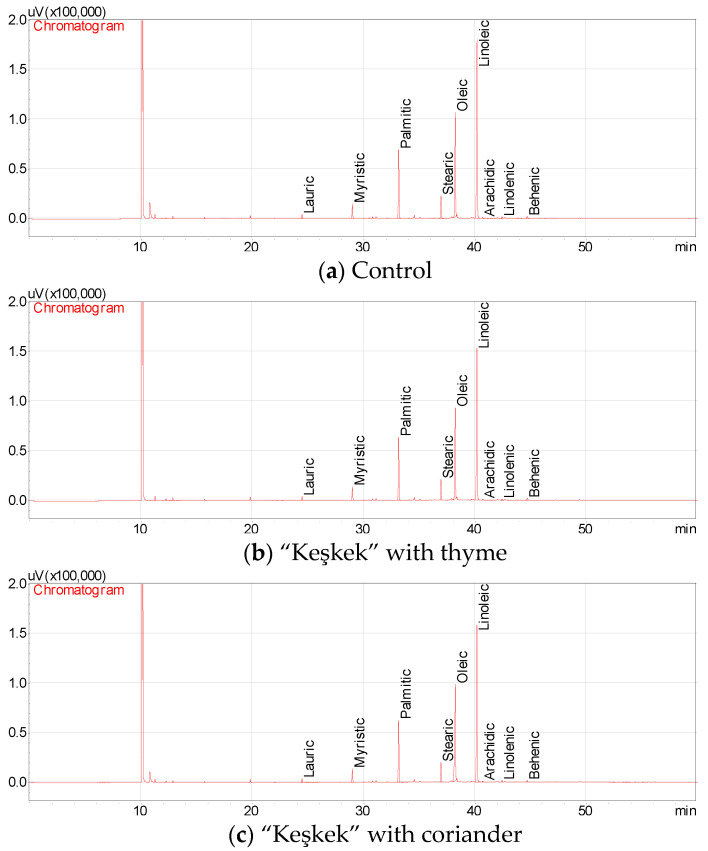

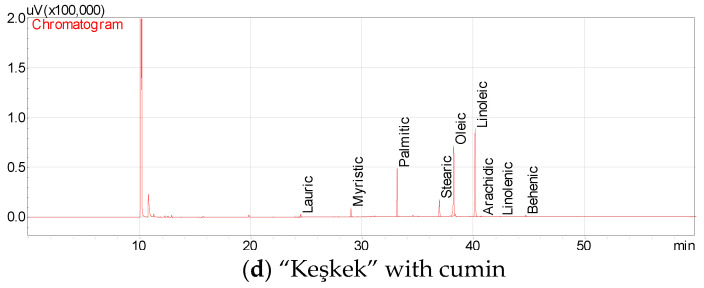
Fatty acid chromatograms of the oils of control and “Keşkek” samples (**a**–**d**) with spice added.

The fatty acid composition and the quantitative values of “Keşkek” samples provided by using thyme, coriander and cumin spice powders are given in Table 3. The dominant fatty acids of “Keşkek” oil samples were myristic, stearic, palmitic, oleic, stearic and linolenic acids (Figure 2). Results changed depending on the addition of spices. Myristic and stearic acids of the oils extracted from “Keşkek” samples were detected between 3.13% (with coriander) and 3.59% (with thyme) to 5.05% (with coriander) and 6.88% (with cumin), respectively. Moreover, the oils of untreated samples and “Keşkek” samples with thyme, coriander and cumin spices contained 15.21%, 16.48%, 14.78% and 18.45% palmitic acids, respectively. While oleic acid amounts of the oils of the “Keşkek” samples were detected as between 25.51% (with thyme) and 30.58% (with cumin), the linoleic acid contents of the oils of “Keşkek” products were identified as between 38.28% (with cumin) and 48.49% (control). The lauric acid amounts of “Keşkek” oils varied between 0.88% (with coriander) and 1.03% (with cumin). The amounts of other fatty acids were found under <0.75% (with cumin). In general, the highest amounts of fatty acids were found in “Keşkek” with cumin, followed by thyme, control (without spice) and “Keşkek” with coriander, in decreasing order. “Keşkek” oils are rich in linoleic acid, followed by oleic, palmitic, stearic and myristic acids, in descending order. Rebey et al. [26] reported that the main fatty acids of the oil of cumin seeds at full maturity were petroselinic acid (55.9%), followed by palmitic (23.82%), linoleic (12.40%) and pamitoleic (2.12%) acids. Coriander vegetable oil contained 2.9% palmitic, 72.7% petroselinic, 6.0% oleic, 13.7% linoleic and 0.2% linolenic acids [27]. Merah et al. [28] reported that cumin seed oils contained 3.9–4.3% palmitic, 47.4–51.6% petroselinic acid, 11.2–12.2% oleic, 30.5–32.9% linoleic and 0.2–0.6% linolenic acids. The results differed from previous fatty acid results with spice fruits or seed oils. These differences may sometimes be due to the fact that the spices are processed into the product and sometimes they are only analyzed in seed oils. Moreover, the differences in the fatty acid components of the “Keşkek” oils may have been due to the added fatty acids in coriander and cumin oil and oils used. This situation is clearly understood from the literature data. The fact that the pancake is rich in linoleic acid shows that this food is also rich in essential fatty acids.

### 3.4. The Mineral Contents of “Keşkek” Samples

Mineral contents of “Keşkek” samples covered with thyme, coriander and cumin spice powders are illustrated in Table 4. P, K, Mg and S were the major minerals of “Keşkek” samples. P and K contents of “Keşkek” samples were detected as between 1466.97 (with cumin) and 1878.08 mg/kg (with thyme) to 1096.73 (with cumin) and 3618.79 mg/kg (with thyme), respectively. Moreover, while Mg amounts of “Keşkek” samples changed to be between 142.49 (with cumin) and 211.26 mg/kg (with coriander), S amounts of “Keşkek” samples were measured as between 361.34 (control) and 457.04 mg/kg (with cumin). In addition, Na and Fe amounts of “Keşkek” varied between 49.98 mg/kg (control) and 65.64 mg/kg (with coriander) to 5.87 mg/kg (with cumin) and 85.32 mg/kg (with coriander), respectively. Mn and Zn contents of “Keşkek” samples were determined between 3.26 mg/kg (with thyme) and 6.18 mg/kg (with cumin) to 3.40 mg/kg (with thyme) and 4.48 mg/kg (with cumin), respectively. The highest B was found in “Keşkek” with coriander (6.92 mg/kg). Ca, Cu and Ni in “Keşkek” samples were found at low levels, and under <1.56 mg/kg. P, K, Ca, Mg and Fe elements were found at their lowest levels in “Keşkek” samples, while the highest P, K and S were found in “Keşkek” with thyme. The P, Mg, S, Na, Mn, Cu and B contents of “Keşkek” with added spices increased when compared to the control. However, Ni contents of “Keşkek” with spices decreased compared to the control. The amounts of other elements have shown differences according to the spices added. While Fe contents of “Keşkek” with coriander were found at the highest level compared to the control, Fe amounts of “Keşkek” with thyme and cumin spices were measured at low levels compared to the control. Potassium is an essential mineral for the proper functioning of the body, body fluid pressure and electrolyte balance. Phosphorus, which works with calcium to build healthy bones in the human body, plays a role in many functions such as filtering wastes in the body and repairing tissues and cells. Most people obtain the amount of phosphorus they need from their daily diet [29]. The differences in the mineral contents of the “Keşkek” samples may have been caused by the mineral amounts of the spices and wheat used.

### 3.5. Sensory Properties of “Keşkek” Samples

Sensory properties of untreated and treated “Keşkek” samples are presented in Table 5. While the color values of the “Keşkek” samples were scored between 4 (coriander) and 5.0 (control, thyme and cumin) by the panelists, the odor values of the “Keşkek” samples were scored between 4 (control, coriander) and 5 (thyme and cumin). In addition, the flavor and texture values of the “Keşkek” samples were scored between 4 (control and coriander) and 5 (thyme and cumin) to 4 (coriander and cumin) and 5 (control and thyme), respectively. Furthermore, the odor and flavor values of thyme- and cumin-containing “Keşkek” samples were more appreciated than the control, while color and texture values were found to be similar. It was observed that the flavor and texture values of “Keşkek” samples with cumin and coriander were slightly lower than the control. When the sensory characteristics of the “Keşkek” samples were taken into account, the “Keşkek” with thyme was favored, followed by the “Keşkek” with cumin, with coriander and the control sample, in descending order. The high appreciation of “Keşkek” with the thyme may be due to the high total phenol and flavonoid content and the highest content of most phenolic compounds.

## 4. Conclusions

It has been determined that “Keşkek” types are effective on the bioactive properties of almond varieties. A linear relation was observed among antioxidant activity and total phenol values (Figure S2). Among these phenolic constituents, gallic acid was the most abundant, followed by catechin, rutin and 3,4-dihydroxybenzoic acid, in descending order. Oleic, linoleic and palmitic acids were the abundant fatty acids of “Keşkek” oils extracted. The gallic acid, 3,4-dihydroxybenzoic acid, catechin and caffeic acid contents of the “Keşkek” sample made with the addition of coriander increased significantly compared to the control, and the amounts of other components decreased. In addition, the phenolic components of “Keşkek” samples made with cumin were significantly increased when compared to the control (except for syringic acid, rutin, p-coumaric acid, quercetin and kaempferol). In general, the highest amounts of fatty acids were found in “Keşkek” with cumin, followed by thyme, control (without spice) and “Keşkek” with coriander, in decreasing order. P, K, Mg and S were the major minerals of “Keşkek” samples. Added spices (cumin and thyme) attracted the attention of the consumers. Therefore, due to the phenolic components of the added spices, the “Keşkek” was liked and consumed by the consumers.

## Figures and Tables

**Table 1:** Some chemical and bioactive properties of “Keşkek” samples.

Keşkek with Spice	Moisture Content (%)	Oil Content (%)	Total Phenolic Content (mg GAE/100 g)	Total Flavonoid Content (mg QE/100 g)	Antioxidant Activity (mmol TE/kg)
Control	7.15 ± 0.34a *	14.90 ± 2.20d	7.02 ± 2.48d	20.24 ± 1.35d	0.04 ± 0.00d
Thyme	5.79 ± 0.05c **	16.05 ± 2.25b	77.10 ± 5.09a	132.14 ± 1.17a	2.78 ± 0.00a
Coriander	5.70 ± 0.27d	15.60 ± 0.40c	23.29 ± 1.57c	64.05 ± 2.43c	0.11 ± 0.00b
Cumin	6.42 ± 0.02b	21.20 ± 0.60a	25.83 ± 2.86b	76.90 ± 4.10b	0.10 ± 0.00bc

* standard deviation; ** values within each column followed by different letters are significantly different at *p* < 0.05.

**Table 2:** Phenolic compounds of “Keşkek” samples (mg/100 g).

Phenolic Compounds	Control	Thyme	Coriander	Cumin
Gallic acid	25.97 ± 0.55d *	26.14 ± 1.21c	26.35 ± 0.52b	50.53 ± 0.34a
3,4-Dihydroxybenzoic	0.40 ± 0.01d **	1.19 ± 0.17c	1.88 ± 0.50b	3.12 ± 0.90a
Catechin	2.54 ± 0.45d	3.16 ± 0.57c	4.99 ± 0.91b	6.52 ± 0.09a
Caffeic acid	0.31 ± 0.05c	0.42 ± 0.01a	0.33 ± 0.01b	0.42 ± 0.04a
Syringic acid	0.41 ± 0.02a	0.22 ± 0.02d	0.32 ± 0.01b	0.24 ± 0.04c
Rutin	2.50 ± 0.36b	4.33 ± 0.23a	2.45 ± 0.23c	1.95 ± 0.29d
*p*-Coumaric acid	0.24 ± 0.03a	0.21 ± 0.02b	0.11 ± 0.01d	0.18 ± 0.03c
Ferulic acid	0.25 ± 0.02c	1.91 ± 0.37a	0.21 ± 0.01d	1.23 ± 0.53b
Resveratrol	0.33 ± 0.10b	0.08 ± 0.01d	0.11 ± 0.01c	1.21 ± 0.07a
Quercetin	1.23 ± 0.37b	1.32 ± 0.17a	0.58 ± 0.10c	0.47 ± 0.05d
Cinnamic acid	0.15 ± 0.04c	0.67 ± 0.13a	0.04 ± 0.01d	0.29 ± 0.09b
Kaempferol	0.23 ± 0.02a	0.19 ± 0.01b	0.12 ± 0.01c	0.12 ± 0.01c

* standard deviation; ** values within each row followed by different letters are significantly different at *p* < 0.05.

**Table 3:** Fatty acid composition of “Keşkek” oils.

Fatty Acids (%)	Control	Thyme	Coriander	Cumin
Lauric	0.89 ± 0.01c *	1.02 ± 0.01b	0.88 ± 0.02d	1.03 ± 0.03a
Myristic	3.21 ± 0.02c **	3.59 ± 0.09a	3.13 ± 0.06d	3.54 ± 0.01b
Palmitic	15.21 ± 0.07c	16.48 ± 0.25b	14.78 ± 0.08d	18.45 ± 0.01a
Stearic	5.36 ± 0.01c	5.60 ± 0.04b	5.05 ± 0.01d	6.88 ± 0.01a
Oleic	25.74 ± 0.04c	25.51 ± 0.14d	29.90 ± 0.07b	30.58 ± 0.00a
Linoleic	48.49 ± 0.07a	46.72 ± 0.15b	45.15 ± 0.09c	38.28 ± 0.01d
Arachidic	0.23 ± 0.00c	0.24 ± 0.00b	0.23 ± 0.00c	0.33 ± 0.01a
Linolenic	0.35 ± 0.00b	0.31 ± 0.00c	0.42 ± 0.00a	0.18 ± 0.00d
Behenic	0.52 ± 0.00b	0.52 ± 0.01b	0.47 ± 0.01c	0.75 ± 0.00a

* standard deviation; ** values within each row followed by different letters are significantly different at *p* < 0.05.

**Table 4:** Mineral content of “Keşkek” samples (mg/kg).

“Keşkek”	P	K	Ca	Mg	S	Na	Fe	Cu	Mn	Ni	Z	B
Control	1460.18 ± 19.23d *	2188.99 ± 169.89b	0.92 ± 0.01b	138.64 ± 4.40d	361.34 ± 16.59d	49.98 ± 1.70d	6.21 ± 0.54b	0.86 ± 0.03d	4.16 ± 0.28b	0.63 ± 0.03a	3.50 ± 0.19c	5.04 ± 0.13d
Thyme	1878.08 ± 6.56a **	3618.79 ± 60.51a	0.75 ± 0.13c	190.25 ± 7.01b	386.26 ± 2.16c	54.10 ± 1.58c	4.59 ± 0.71c	1.08 ± 0.07c	3.26 ± 0.38c	0.57 ± 0.02b	3.40 ± 0.45d	5.94 ± 0.12c
Coriander	1516.26 ± 42.96b	2112.09 ± 258.22c	1.05 ± 0.02a	211.26 ± 4.69a	437.36 ± 17.26b	65.64 ± 3.99a	85.32 ± 0.42a	1.36 ± 0.08b	6.17 ± 0.12a	0.44 ± 0.02d	3.76 ± 0.08b	6.92 ± 0.12a
Cumin	1466.97 ± 14.31c	1096.73 ± 118.99d	0.43 ± 0.06d	142.49 ± 30.54c	457.04 ± 114.72a	58.35 ± 0.99b	0.42 ± 0.96d	1.56 ± 0.12a	6.18 ± 0.48a	0.52 ± 0.02c	4.48 ± 0.58a	6.57 ± 0.11b

* standard deviation; ** values within each column followed by different letters are significantly different at *p* < 0.05.

**Table 5:** Sensory properties of Keşkek samples.

	Control	Thyme	Coriander	Cumin
Color	5 ± 0.20a *	5 ** ± 0.15a	4 ± 0.20b	5 ± 0.10a
Odor	4 ± 0.75b	5 ± 0.20a	4 ± 0.50b	5 ± 0.50a
Flavor	4 ± 0.25b	5 ± 0.25a	4 ± 0.75b	5 ± 0.25a
Texture	5 ± 0.25a	5 ± 0.20a	4 ± 0.20b	4 ± 0.75b
General appreciation	4.5	5.0	4.0	4.75

* standard deviation; ** values within each row followed by different letters are significantly different at *p* < 0.05.

## Data Availability

The data presented in this study are available within the article.

## Supplementary Materials

The following are available online at https://www.mdpi.com/article/10.3390/foods11213492/s1, Figure S1: Curve and chromatograms for phenolic compounds, Figure S2: Relation between total phenol contents and antioxidant activity of “Keşkek” samples.
