# Supplementary material for: The Effect of Spice Powders on Bioactive Compounds, Antioxidant Activity, Phenolic Components, Fatty Acids, Mineral Contents and Sensory Properties of “Keşkek”, Which Is a Traditional Food
Source: Foods. 2022 Nov 3;11(21):3492. doi: 10.3390/foods11213492 (PMC9654948; doi:10.3390/foods11213492)
Supplement: Supplementary file 1 [file foods-11-03492-s001.zip › foods-2013028-supplementary.pdf]

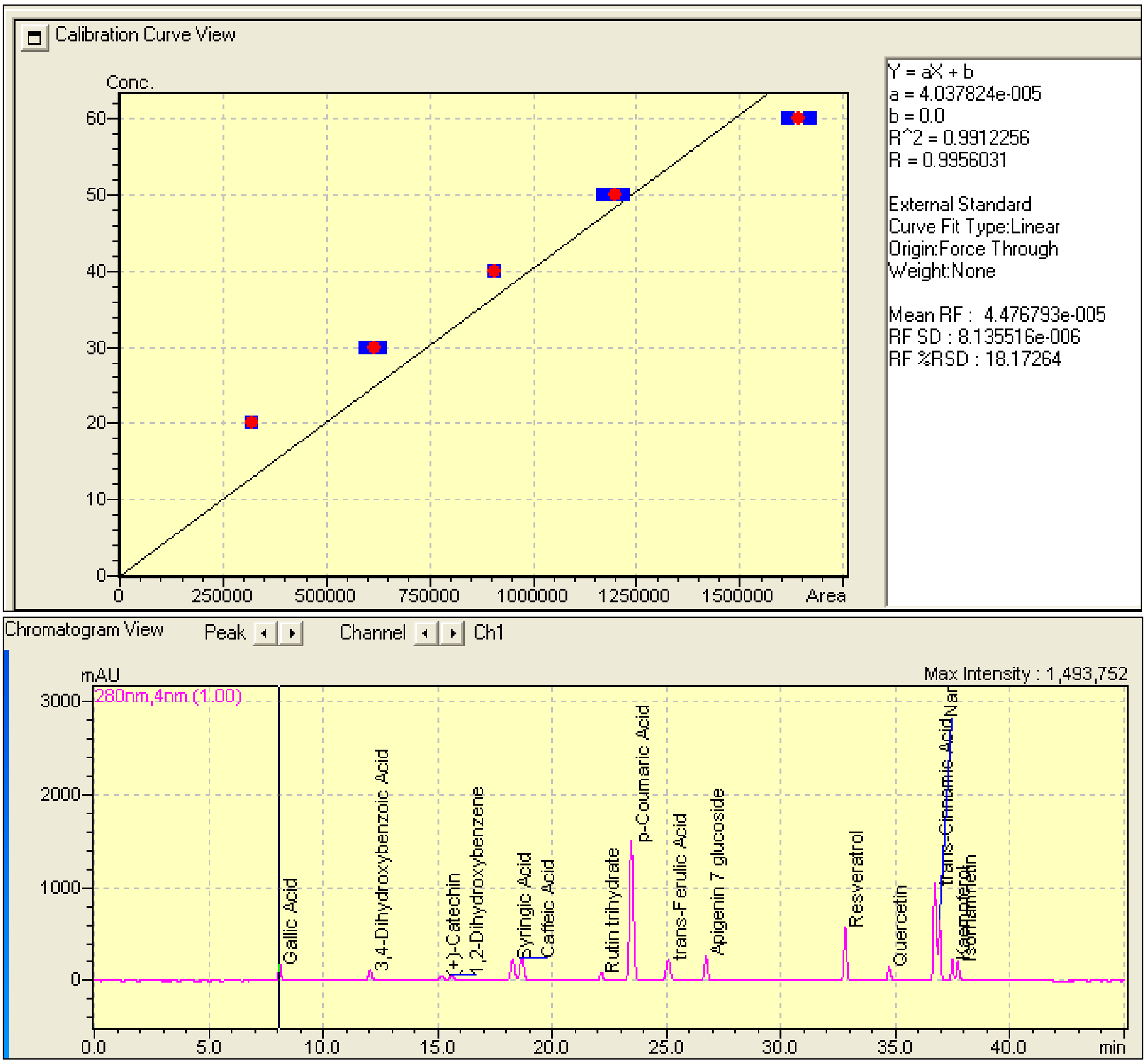

Figure S1. Curve and chromatograms for phenolic compounds.

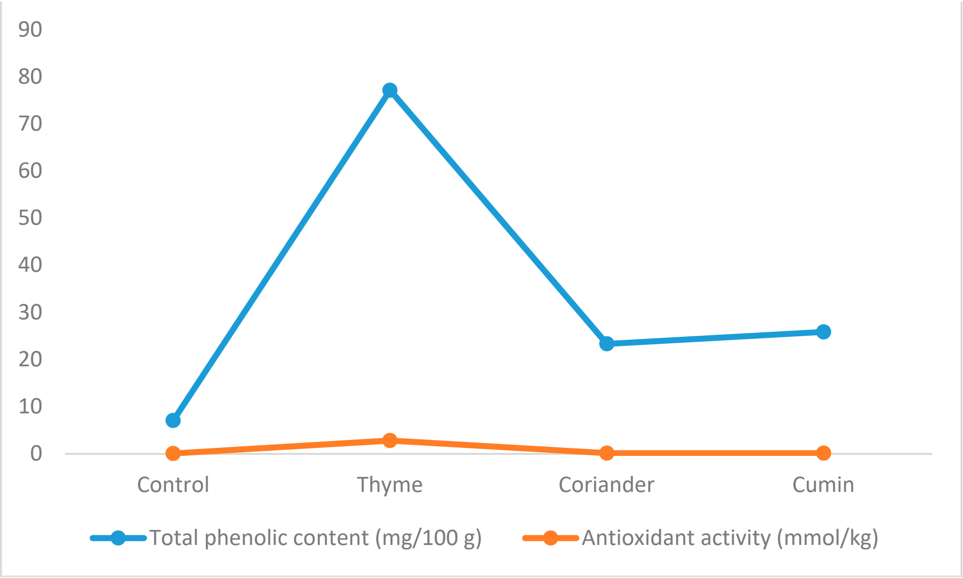

Figure S2. Relation between total phenol contents and antioxidant activity of "Keşkek" samples.
